# Methicillin-sensitive Staphylococcus aureus lineages contribute towards poor patient outcomes in orthopaedic device-related infections

**DOI:** 10.1099/mgen.0.001390

**Published:** 2025-04-16

**Authors:** Virginia Post, Ben Pascoe, Matthew D. Hitchings, Christoph Erichsen, Julian Fischer, Mario Morgenstern, R. Geoff Richards, Samuel K. Sheppard, T. Fintan Moriarty

**Affiliations:** 1 AO Research Institute Davos, Davos, Switzerland; 2 Ineos Oxford Institute for Antimicrobial Research, Department of Biology, University of Oxford, Oxford, UK; 3 Faculty of Veterinary Medicine, Chiang Mai University, Chiang Mai, Thailand; 4 School of Animal and Comparative Biomedical Sciences, University of Arizona, Tucson, Arizona, USA; 5 Swansea University Medical School, Swansea University, Swansea, UK; 6 Department of Trauma Surgery, Trauma Centre Murnau, Murnau, Germany; 7 Centrum of Orthopedic Isartal, Pullach im Isartal, Germany; 8 Department of Orthopedic and Trauma Surgery, University Hospital, Basel, Switzerland

**Keywords:** antibiotic resistance, methicillin-resistant *Staphylococcus aureus* (MRSA), methicillin-susceptible *Staphylococcus aureus* (MSSA), orthopaedic device-related infections, *Staphylococcus aureus*, virulence factors

## Abstract

Staphylococci are the most common cause of orthopaedic device-related infections (ODRIs), with *Staphylococcus aureus* responsible for a third or more of cases. This prospective clinical and laboratory study investigated the association of genomic and phenotypic variation with treatment outcomes in ODRI isolates. Eighty-six invasive *S. aureus* isolates were collected from patients with ODRI, and clinical outcome was assessed after a follow-up examination of 24 months. Each patient was then considered to have been ‘cured’ or ‘not cured’ based on predefined clinical criteria. Whole-genome sequencing and molecular characterization identified isolates belonging to globally circulating community- and hospital-acquired lineages. Most isolates were phenotypically susceptible to methicillin and lacked the staphylococcal cassette chromosome *mec* cassette [methicillin-susceptible *S. aureus* (MSSA); 94%] but contained several virulence genes, including toxins and biofilm genes. Whilst recognizing the role of the host immune response, we identified genetic variance, which could be associated with the infection severity or clinical outcome. Whilst this and several other studies reinforce the role antibiotic resistance [e.g. methicillin-resistant *S. aureus* (MRSA) infection] has on treatment failure, it is important not to overlook MSSA that can cause equally destructive infections and lead to poor patient outcomes.

**Impact Statement**

*Staphylococcus aureus* is a prominent cause of orthopaedic device-related infections (ODRI), yet little is known about how the infecting pathogen and specifically the repertoire of genome-encoded virulence factors can impact treatment outcomes. Past studies have focused on distinguishing commensal from invasive *S. aureus* isolates, but in this study, we aim to investigate traits in infecting isolates that influence patient outcomes. Invasive *S. aureus* isolates were collected between November 2011 and September 2013 from ODRI patients and categorized according to the success of subsequent treatment (‘cured’/‘not cured’), as determined by a follow-up 2 years after initial presentation. Several methicillin-susceptible *S. aureus* clones were associated with a ‘not cured’ clinical outcome. An improved understanding of the bacterial traits associated with treatment failure in ODRI will inform the risk assessment, prognosis and therapy of these infections.

## Data Summary

Illumina short read sequence data are archived on the Sequence Read Archive associated with BioProject accession PRJNA529795. Assembled genomes and supplementary material, including analysis output files, are shared on figshare (doi: 10.6084/m9.figshare.7926866) and are available on our public Staphylococcal Bacterial Isolate Genome Sequence Database: https://sheppardlab.com/resources/. Isolate genealogy with associated meta-data is visualized on Microreact [1]: https://microreact.org/project/post-pascoe-odri-saureus. All high-performance computing was performed on MRC CLIMB [2] in a bioconda environment [3].

## Introduction

The most challenging complication in orthopaedic surgery is orthopaedic device-related infection (ODRI), with incidence ranging from 0.7% to 4.2% for elective orthopaedic surgeries [4,7]. Incidence increases to over 30% following the operative fixation of complex open lower leg fractures [8,9]. Whilst patient health (including a high body mass index and chronic immunosuppression) is a key risk factor for poor treatment outcomes [10,11], there is evidence that pathogen genetic diversity can be an indicator of patient outcome [12,15]. *Staphylococcus aureus* is the most common infecting agent [5,16,19], and treatment outcome is often complicated by infection with antimicrobial-resistant lineages, e.g. methicillin-resistant Staphylococci [20,21]. Combined with high virulence potential, methicillin-resistant *S. aureus* (MRSA) infections are difficult to treat and are a global healthcare concern. Despite this, predicting treatment outcomes based on bacterial phenotypes/genotypes remains difficult [11,13, 14, 22].

Clonal lineages have helped spread *S. aureus* around the world, and their geographic distribution is dynamic, with different clones and sequence types (STs) dominating infection cases in specific global regions [23,24]. Waves of MRSA have risen and been replaced since the emergence of MRSA in the 1940s [23,25]. These highly structured populations can be grouped into clonal complexes (CCs) that share five or more alleles at seven multilocus sequence typing (MLST) loci [26,28]. Lineages that have been characterized as predominantly community-associated MRSA (CA-MRSA) have begun to replace hospital-associated MRSA (HA-MRSA) as the dominant epidemic strains [29,30]. The most prevalent lineages include five global community-acquired (CA-MRSA) genotypes CC1, CC8, CC30, CC59 and CC80. The most common hospital-acquired lineages are CC5, CC22 (UK) and CC45 [26,28]. Combining MLST with traditional molecular typing techniques such as identification of the staphylococcal cassette chromosome *mec* (SCC*mec*) [31] and *spa* repeat regions [32] can provide a nomenclature to describe relevant epidemic clones. Lineages can acquire advantageous traits, such as antibiotic resistance, which proliferate in the population through the decedents of successful strains [33]. This likely occurs in many instances; however, the extent of this in the context of ODRI remains to be determined.

Despite the growing concern of MRSA lineages, methicillin-susceptible *S. aureus* (MSSA) isolates are often the most common in invasive surgery-related infections [34,35]. Convergent evolution in several CA lineages potentially balances the fitness costs of expressing antimicrobial resistance (AMR) genes with the acquisition of multiple virulence factors [36,39]. Specific genes encoding putative virulence factors in invasive *S. aureus* disease involve evasion of immune defences, including the ability to adhere to and invade host tissues – essential for ODRI [40,41]. A large body of work has identified many virulence factors, including microbial surface components recognizing adhesive matrix molecules, the polysaccharide intercellular adhesion, the staphylococcal protein A, extracellular proteins such as coagulase, staphylococcal enterotoxins (SEs), exfoliatins, toxic shock syndrome toxin (TSST), staphyloxanthin, haemolysins, Panton–Valentine leukocidin (PVL) and proteins encoded by genes belonging to the immune evasion cluster (IEC) that have a crucial impact on the pathogenicity of *S. aureus* infections [42,48]. Genome-wide association (GWAS) studies have been applied to numerous bacterial species [49,50] and identified genes or genetic elements associated with disease that transcend clonal patterns of inheritance (not confined to specific lineages) in staphylococci. Phenotype filtering techniques have attempted to assess patient risk and predict disease outcome from genetic data based on enrichment for disease-associated traits [15,51,53]. GWAS approaches have shown promising disease prediction results [38,53, 54], as well as AMR profiles [55]. As in other bacterial species [56,58], a better understanding of genome and transcriptome variation in infection types shows promise for our ability to predict disease severity in staphylococci [59].

In this study, we investigate the association between treatment outcomes determined 2 years post-operatively in patients with * S. aureus* ODRI (isolated between November 2011 and September 2013) and phenotypic and genotypic features of the infecting pathogen. Building on recent studies aiming to predict *S. aureus* virulence from genome sequence [53,54], we aim to distinguish high-risk lineages, isolates and genes. These features were correlated with the mortality rate in a simplified virulence model using *Galleria mellonella*.

## Methods

### Sampling context

The clinical data and *S. aureus* collection were part of a prospective study performed between November 2011 and September 2013 at BGU Murnau in Germany [15], which was approved by the local ethical committee *Ethik-Kommission der Bayerischen Landesärztekammer* under approval number 12063 and registered with https://clinicaltrials.gov with identifier NCT02971657. The BG Unfallklinik Murnau serves as one of Germany’s premier trauma and rehabilitation centres, specializing in severe injuries, particularly for patients covered by the German Statutory Accident Insurance. Whilst it primarily serves southern Bavaria, its specialized services attract patients from across Germany. It is a leader in treating spinal cord injuries, severe burns and (importantly for our study) multiple trauma cases. Patients often travel from other regions and countries due to the clinic’s advanced capabilities in areas such as acute care, long-term rehabilitation and highly specialized reconstructive surgeries. The hospital has a level I trauma centre, with a dedicated department for septic and reconstructive surgery with 40 beds.

### Patient enrolment and surgical sampling

All patients enrolled in the study were aged 18 or older and provided informed written consent. Patient inclusion criteria were culture-positive *S. aureus* infection involving fracture-related infection (FRI) or peri-prosthetic joint infections (PJIs) as previously defined [11,15, 60, 61]. Briefly, four criteria needed to be met, including (1) the presence of fistula, sinus or wound breakdown; (2) purulent drainage; (3) phenotypically undistinguishable pathogens identified from two or more cultures; and (4) the presence of micro-organisms from deep tissue during operative intervention. No time limit was imposed on the period between device implantation and the onset of infection. Three biopsies were taken from three distinct sites within the surgical field. At least two biopsies were required to be positive for confirmation of infection [62,64]. A single implant infection isolate was collected from each patient during surgical intervention, and patient outcomes were measured. Pre-operative colonization data were not routinely collected for the patients in this study. Whilst this may be possible for elective patients where surgery is planned, many of our patients were trauma patients, where pre-operative screening is impossible, and not standard practice for emergency patients in Germany.

During the first surgical procedure performed after enrolment, bone biopsies were taken from the interface between the implant and the affected bone. Samples were placed in a sterile container with thioglycolate liquid medium (bioMérieux, Hazelwood, MO, USA) and cultured for 10 days at 37 °C. Any growth was sub-cultured onto a blood agar plate (bioMérieux, Hazelwood, MO, USA) for subsequent identification. All isolates were grown on tryptone soy agar (TSA, Oxoid, Pratteln, Switzerland) and incubated overnight at 37 °C. Species identification was performed using a Vitek2 (bioMerieux Vitek Inc., Hazelwood, MO). A single colony was then taken and resuspended in 1 ml tryptone soy broth (TSB, Oxoid, Pratteln, Switzerland) containing 20% vol/vol glycerol for long-term storage at −80 °C. Infections with other *Staphylococcus* species or polymicrobial infections were excluded.

### Clinical management and patient follow-up

Patient risk data were collected at the time of the initial operation, all patient details were anonymized and *S. aureus* isolates were given genome database identifiers (BIGSids), study identifiers (ARI-number) and sample laboratory identifiers (Lab-ID). Associated clinical data are summarized in Table S1 (available in the online Supplementary Material). All patients were managed in a consistent manner based on the treatment principles of this institution. Typically, patients receive serial debridement until two consecutive intraoperative cultures are negative, at which time pathogen-adapted antibiotic therapy is administered. After an average of 23 months of follow-up, clinical outcomes were assessed by an orthopaedic surgeon. ‘Cured’ patients were free of infection, previously defined as including symptom resolution, normalization of inflammatory markers and clinical judgement, and no requirement for additional revision surgery during the intervening period – surgical and systemic antibiotic therapy – had ceased with the function of the affected joint or limb restored. If one or more of these parameters were negative, patients were considered to have had a ‘not cured’ outcome [11,15]. There are ‘not cured’ outcomes that were not based on the persistence of infection – a poor outcome may have been amputation, for example, where the infection led to drastic surgical intervention. Similarly, a patient may have a ‘cured’ outcome that was due to extensive debridement, and the bacteria were all surgically cut out from the wound, meaning that there was no possibility for recurrence of infection regardless of pathogen virulence.

### Genome sequencing and assembly

*S. aureus* colonies were cultured in 5 ml tryptone soy broth (TSB, Oxoid AG, Pratteln, Switzerland) at 37 °C with overnight shaking. DNA was extracted using the QIAamp DNA Mini Kit (Qiagen, Germany) according to the manufacturer’s instructions with the addition of 1.5 µg µl^−1^ lysostaphin (Sigma-Aldrich, Buchs, Switzerland) and 2 µg ml^−1^ lysozyme (Sigma-Aldrich, Buchs, Switzerland) to facilitate cell lysis. Whilst the addition of lysozyme is not essential for the extraction of DNA from *S. aureus*, laboratory protocols were followed to permit extraction from multiple *Staphylococcus* species simultaneously. DNA was quantified using a spectrophotometer prior to sending for sequencing by Microsynth AG (Switzerland) using an Illumina MiSeq benchtop sequencer. Sequencing libraries were prepared using Nextera XT library preparation kits (v2) and paired-end 250 bp reads generated with the MiSeq run kit (v2). Short-read paired-end data were assembled *de novo* with SPAdes (version 3.3.0; using the *–careful* command) [65] and assessed for quality (*n*=86 genomes contained <500 contigs). Average assembled genomes were 2,771,938 bp in length (range: 2,638,312–2,962,075 bp) consisting of 113 contigs (range: 29–358 contigs) with an N50 of 43,492 bp (range: 5,729–109,980) (Table S2).

### Genome archiving, multiple genome alignments and construction of isolate genealogies

An alignment of all 86 *S*. *aureus* isolates was constructed from concatenated gene sequences of all core genes (found in ≥95% isolates) using MAFFT (version 7) [66] on a gene-by-gene basis (size: 2,138,455 bp). A maximum-likelihood phylogeny was constructed using a general time reversible substitution model (GTR+I+G) and ultra-fast bootstrapping (1,000 bootstraps) implemented in IQ-TREE (version 2.0.3) [67,69] and visualized on Microreact: https://microreact.org/project/post-pascoe-odri-saureus [1]. Genome collection has also been shared on the pathogen watch website (https://pathogen.watch/collection/7rgjyrzz3xoc-post-and-pascoe-et-al-2022-hypervirulent-mssa-from-odri).

### Molecular typing and genome characterization

Our publicly available Bacterial Isolate Genome Sequence Database (https://sheppardlab.com/resources/) includes functionality to determine MLST profiles defined by the Staphylococcal pubMLST database (https://pubmlst.org/saureus; accessed March 2019) [70]. Staphopia (version 1.0.0) was used to define SCC*mec* types [71], and *spa* types were typed *in silico* using spaTyper v1.0 (Table S2) [72].

### Characterization of the core and accessory genomes

All unique genes present in at least one of our isolates (or eight common *S. aureus* reference strains, Table S2) were identified by automated annotation using Prokka (version 1.13 [73]) followed by PIRATE [74], a tool that allows for orthologue gene clustering in bacteria (Table S3). Gene families in PIRATE were defined using a wide range of aa percentage sequence identity thresholds for Markov Cluster algorithm clustering (45, 50, 60, 70, 80, 90, 95 and 98). Core genes were defined as present in 95% of the genomes and accessory genes as present in at least one isolate (Fig. S1A). The pangenome was visualized using phandango, as a matrix of gene presence alongside a core genome phylogeny [75]. Pairwise core and accessory genome distances were compared using PopPunk (version 1.1.4 [76]), which uses pairwise nt k-mer comparisons to distinguish shared sequence and gene content to identify divergence of the accessory genome in relation to the core genome. A two-component Gaussian mixture model was used to construct a network to define clusters and visualized with Microreact (Fig. S1BC) [1,76].

### Identification of known virulence genes and antimicrobial resistance genes (ARGs)

The presence of putative virulence genes and antibiotic resistance genes (ARGs) was identified from assembled genomes using ABRICATE (v0.3) [76]. Putative virulence genes were detected through comparison with reference nt sequences in the virulence finder database (VfDb; default settings: ≥70% identity over ≥50% of the gene; Tables S4 and S5 [77]. ARGs were identified using the NCBI AMRFinderPlus [78] and CARD [79] databases (19 April 2020 update). Results for both AMR databases were similar, with the CARD database identifying additional AMR-associated genes. We report the results from the curated AMRFinderPlus database (Table S6).

## Phenotype testing

### Antibiotic susceptibility testing

Minimum inhibatory concentrations (MICs) for 29 antibiotics were determined using a Vitek2 machine (bioMérieux Vitek Inc., USA) as described previously [11,15]. Susceptibility breakpoints were defined according to the definitions of the European Committee of Antimicrobial Susceptibility Testing, and isolates resistant to three or more antimicrobial classes were defined as multidrug-resistant (MDR) [80]. The Vitek2 results for oxacillin and cefoxitin were used as a proxy for methicillin resistance. Where there was a discrepancy between results, the cefoxitin result was used due to reports of higher sensitivity as a proxy for methicillin resistance [81].

### Staphyloxanthin production

The production of staphyloxanthin is indicated by a yellow-orange pigmentation of *S. aureus* colonies when grown on basic medium agar containing 1% tryptone (Oxoid AG, Pratteln, Switzerland), 0.5% yeast extract (Sigma-Aldrich, Buchs, Switzerland), 0.5% NaCl (Sigma-Aldrich, Buchs, Switzerland), 1.5% agar bacteriology (Oxoid, AG, Pratteln, Switzerland), 0.1% K_2_HP0_4_ (Sigma-Aldrich, Buchs, Switzerland) and 0.1% glucose (Sigma-Aldrich, Buchs, Switzerland) [82]. Isolates were incubated at 37 °C for 48 h [82]. Staphyloxanthin production can be divided into three groups: yellow-orange (strong production), yellow (weak production) and white (absent). All isolates were visually evaluated against reference strains (USA300, COL and ATCC25895) by two independent observers. In this study, strong and weak production was combined into staphyloxanthin producer and white colonies into no staphyloxanthin producer [82].

### Haemolytic activity

*S. aureus* uses a suite of secreted proteins to combat host defences and colonize infection sites. As a proxy for this, we assayed the haemolytic activity of the *S. aureus* isolates by observing their ability to achieve a clear ring of erythrocyte lysis around the colony on blood agar [82]. This phenotypic assay does not differentiate between various haemolytic toxins, which may involve alpha-, beta- or gamma-toxins, as well as phenol-soluble modulins [83]. Briefly, single colonies were plated on sheep blood agar (Oxoid AG, Pratteln, Switzerland) containing 5% vol/vol defibrinated sheep blood (Oxoid AG, Pratteln, Switzerland) and incubated at 37 °C for 24 h followed by cold shock at 4 °C for 12 h. In this way, erythrocyte hydrolysis on sheep blood agar plates is observed, indicated by a clearing zone around the colonies. Isolates were scored into either haemolytic active or non-active [82].

### Biofilm formation

The formation of biofilm was assayed as described previously [11,15, 84, 85]. Briefly, overnight cultures grown in tryptone soy broth (TSB, Oxoid AG, Pratteln, Switzerland), suspended 1 : 100 in fresh TSB containing 1% glucose (Sigma), were incubated in flat-bottomed 96-well tissue culture-treated polystyrene microtitre plates (Nuclon, Nunc A/S, Denmark) for 24 h at 37 °C. Plates were rinsed with phosphate-buffered saline (PBS,Sigma-Aldrich, Buchs, Switzerland), dried and stained with 150 µl of Gram’s crystal violet solution (Sigma-Aldrich, Buchs, Switzerland). Plates were rinsed again with tap water and air-dried. The dye bound to the cells was solubilized by the addition of 150 µl of 95% ethanol. Optical density (OD) was measured as absorbance at 595 nm using a microtitre plate reader (Multiskan GO, Thermo Scientific). All isolates were tested in triplicate in three independent experiments (Table S7). Each microtitre plate also consisted of negative controls (wells without bacterial inoculation) to determine background OD. The results were evaluated using the scale described by Morgenstern *et al.*, Post *et al.* and Stepanovic *et al*. [11,15, 84, 85]. The optical density cut off (ODc) for biofilm formation was determined as the average OD of the negative control+3×sd of the negative control. The four categories – no biofilm formation ability (0; OD≤ODc), weak biofilm formation (1; ODc<OD≤2×ODc), moderate biofilm formation (2; 2×ODc<OD≤4×ODc) and strong biofilm formation ability (3; OD>4×ODc) – were combined in this study into biofilm producer (weak, moderate and strong) and no biofilm producer.

## Statistical analysis

The association amongst and between the clinical parameters, bacterial phenotypes, clades and presence/absence of genes were analysed statistically using the chi-square test. The Mann–Whitney U test was employed to compare differences between genes in the core genome and identified virulence genes. Statistical analyses were performed using SPSS (version 23, IBM, USA) or GraphPad Prism 6 (GraphPad Software, Inc.; Table S8).

### *In vivo* virulence and survival assay in *G. mellonella* larvae

The invertebrate *G. mellonella* infection model has been used effectively to study the virulence of the *S. aureus* strains, with protocols previously described [86,87]. *G. mellonella* were obtained at the pre-larval stage (Entomos AG, Zurich, Switzerland). Larvae were grown at 30 °C in the dark, and groups of ten larvae in the final instar larval stage weighing 200–400 mg were used in all assays. A bacterial suspension of 10^6^ colony forming units (cfu) ml^−1^ was prepared. Quantitative culture of a sample from each bacterial suspension was performed immediately after preparation by tenfold serial dilution and plating on TSA plates to check the actual total viable count of the prepared suspension. Bacterial inoculates (10 µl) were injected into the last left pro-leg into the hemocoel of the last-instar larvae (200–400 mg). After injection, larvae were incubated at 37 °C in the dark. Larvae were assessed daily for survival up to 5 days post-injection and were evaluated according to survival, being scored as dead when they displayed no movement in response to touch. Controls included a group of larvae that did not receive any injection and a group of larvae inoculated with sterile PBS. Experiments consisted of ten larvae per bacterial strain, which was repeated in three separate experiments. For *G. mellonella* survival analysis, larvae mean that survival curves were plotted using the Kaplan–Meier method (GraphPad Prism 6, USA; Table S8).

### Pangenome-wide association studies of phenotype variation

Pangenome-wide differences in gene presence were quantified using the GWAS software, Scoary (version 1.6.14) [88]. With only limited numbers of samples in our collection, we report phylogenetically naïve differences in gene presence between clinical and laboratory symptoms and phenotypes (Table S9). The number of polymorphisms introduced by mutation and recombination in the core genome was inferred using gubbins (version 2.4.1) [89] for each isolate (per branch; Table S10). The consistency of the phylogenetic tree to patterns of variation in sequence alignments for each gene of interest was calculated as before [90, 91]. Consistency indices for each single-gene alignment of 130 virulence-associated genes to a phylogeny constructed using an alignment of 2150 core genes shared by 86 isolates were calculated using the ci function of the R phangorn package [92].

## Results

*S. aureus* isolates were collected from 86 patients undergoing operative revision of an ODRI (Table S1), and patient outcomes were assessed after a 2-year follow-up period. ‘Cured’ patients were free of infection, and surgical and systemic antibiotic therapy had ceased with the function of the affected joint or limb restored [11,15]. Most patients enrolled in the study received successful treatment and were amongst the ‘cured’ cohort (Table 1; *n*=65/86; 75.6%). Treatment was unsuccessful in 21 patients (*n*=21/86; 24.4% ‘not-cured’), and multiple revision surgeries were necessary for nearly all patients (*n*=83/86; 96.5%).

**Table 1.** Cohort description

		Complete study cohort		
Total, *n* (%)	86	(100%)		
	Yes		No	
	*n*	(%)	*n*	(%)
***Infection characteristics***				
PJI	19	22.1	67	77.9
FRI	67	77.9	19	22.1
Acute infection* (in FRI and PJI)	25	29.1	61	70.9
FRI following an initially open fracture	21	24.4	65	75.6
***Health status***				
Obesity	25	29.1	61	70.9
Smoking	28	32.6	58	67.4
Diabetes	16	18.6	70	81.4
Chronic immunosuppression	11	12.8	75	87.2
***Type of implant***				
Peri-prosthetic joint	19	22.1	67	77.9
Nail	19	22.1	67	77.9
Plate	36	41.9	50	58.1
Other small implants†	12	14.0	74	86.0
***Clinical course and infection outcome***				
Multiple revision surgeries	83	96.5	3	3.5
Cured	65	75.6	21	24.4

* Acute infection is defined as an infection with a duration of symptoms of up to 3 weeks.

† The group ‘other small implants’ includes screws (*n*=6), external fixators (*n*=4), a K-wire (*n*=1) and a ligament reconstruction device (*n*=1).

### Host-associated risk factors

Extensive patient data, types of implanted devices and clinical presentation were recorded for each infected patient (Table 1). Additionally, the effects of patient co-morbidities (such as diabetes or obesity), fracture types or the time of symptom onset on treatment outcome were analysed (Table 2). None of these prognostic factors alone significantly decreased the cure rate.

**Table 2.** Patient risk factors

		Cured outcome				
Prognostic factor		No		Yes		***P***-value*
		*n*	(%)	*n*	(%)	
**Total**		21	24.4	65	75.6	
***Obesity***						0.295
	No	13	21.3	48	78.7	
	Yes	8	32.0	17	68.0	
***Smoking***						0.931
	No	14	24.1	44	75.9	
	Yes	7	25.0	21	75.0	
***Diabetes mellitus***						0.559
	No	18	25.7	52	74.3	
	Yes	3	18.8	13	81.3	
***Chronic immunosuppression***						0.606
	No	19	25.3	56	74.7	
	Yes	2	18.2	9	81.8	
***Open fracture (initially)***						0.510
	No	17	26.2	48	73.8	
	Yes	4	19.0	17	81.0	
***Acute infection***						0.541
	No	16	26.2	45	73.8	
	Yes	5	20.0	20	80.0	
***Multiple revision surgeries***						0.316
	No	0	0.0	3	100.0	
	Yes	21	25.3	62	74.7	

* Chi-square test; a *P*-value<0.05 was considered significant.

### Multiple lineages contribute to poor patient outcomes

**Fig. 1.**
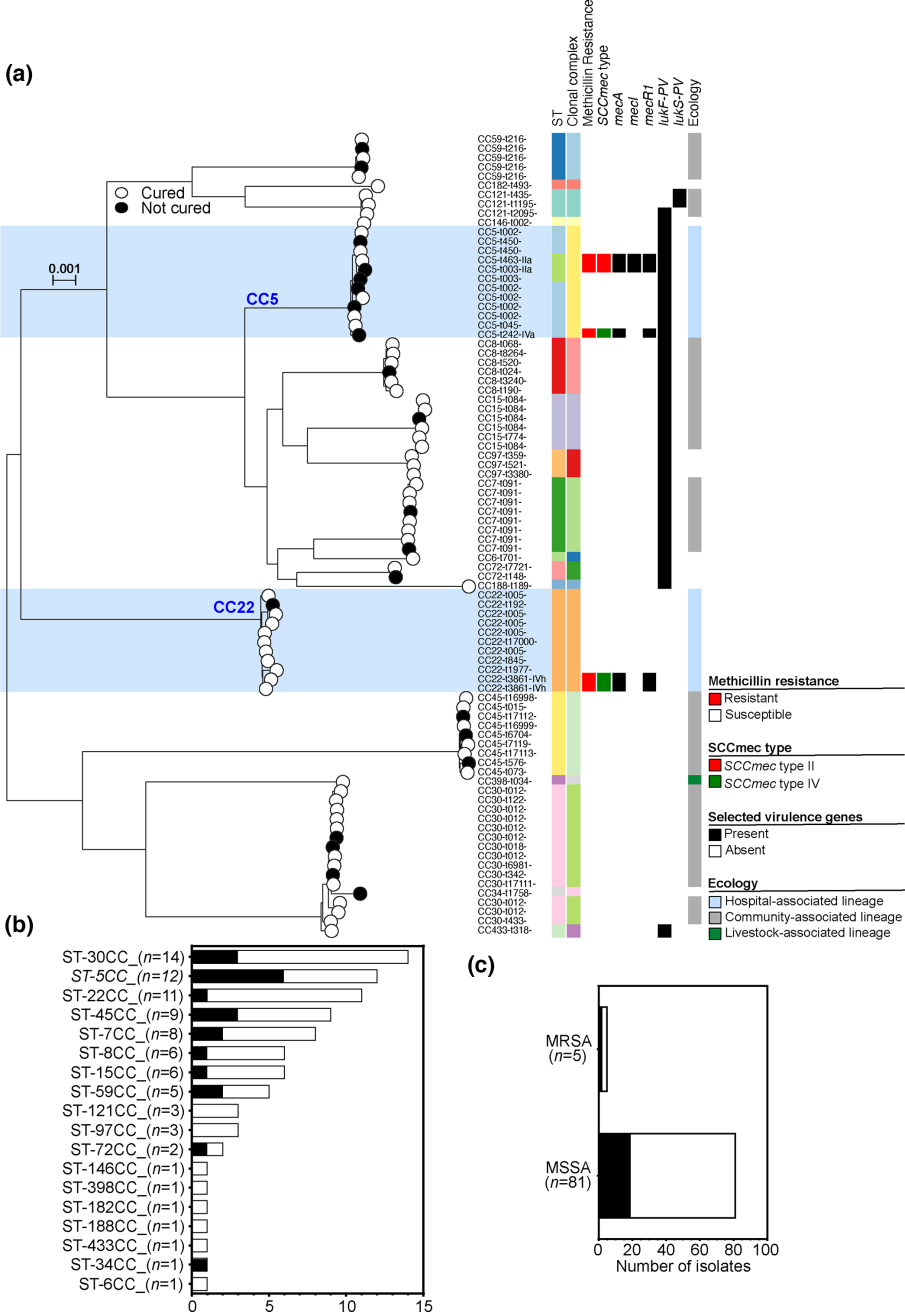
Population structure of *S. aureus* isolates collected in this study. (a) A maximum-likelihood phylogeny was constructed with IQ-TREE, using a GTR model and ultrafast bootstrapping (1,000 bootstraps; version 2.0.3) [67,68] from an alignment of all isolates (*n*=86). The scale bar represents a genetic distance of 0.001. Leaves from isolates that were ‘cured’ are white (*n*=65), and those that did not achieve a ‘cured’ status are black (*n*=21). The tree is annotated with MLST, CC, methicillin resistance status, SCC*mec* and *spa* types and the presence of PVL genes (indicated by coloured blocks). Lineages have been characterized as predominantly HA-MRSA (highlighted in blue), including CC5, CC22 and CC45, and globally spread CA-MRSA genotypes CC1, CC8, CC30, CC59 and CC80. Interactive visualization is available on Microreact [1]: https://microreact.org/project/post-pascoe-odri-saureus. The number of isolates from each (b) MLST CC and (c) methicillin-resistant (MRSA) and methicillin-susceptible (MSSA) lineages. The proportion of isolates that lead to a ‘not cured’ status is shown in black.

A maximum-likelihood phylogeny was constructed based on shared coding sequences, present in 95% or more isolates (Fig. 1a, Table S1). The collected isolates were genetically diverse and represented 19 different STs (Fig. 1b) based on the seven loci scheme for * S. aureus* and could be grouped into 18 CCs based on five or more shared MLST loci [26]. No clear clustering was observed between ‘cured’ and ‘not-cured’ isolates, with isolates from ‘cured’ cases (*n*=65) assigned to 18 different STs (Simpson’s diversity index=0.0819; 96% CI: 0.706–0.932), and isolates from ‘not-cured’ cases (*n*=21) found in 11 different STs (Simpson’s diversity index=0.719; 95% CI: 0.578–0.860). Consistent with other European surveillance efforts, six of our seven most common lineages matched common globally dispersed *S. aureus* lineages (CC5, CC8, CC22, CC30, CC45 and CC59) previously identified by the ESCMID study group (collected from 26 European countries between 2006 and 2007) [93]. Our isolates predominantly lacked the SCC*mec* cassette, which confers methicillin resistance, and were classified as MSSA (*n*=81/86; 94%) (Fig. 1c).

Most of our isolates (*n*=48/86; 55.8%) were from lineages most often acquired in the community (CA-MSSA or SCC*mec* types I, II and III), based on previous reports [36,37, 94]. CC30 was the most common CC identified in our collection (*n*=14/86; 16.3%) (Fig. 1a, b). In more than three quarters (*n*=11/14; 78.6%) of cases where this CC was identified, the patient was deemed to have had a good outcome and a ‘cured’ status (Table S2). A minority of isolates were identified from other CA lineages: CC8 (*n*=6), CC59 (*n*=5), CC45 (*n*=9), CC7 (*n*=8) and CC15 (*n*=6). No isolates were sampled from two common pandemic CA lineages: CC1 and CC80.

Many isolates that we identified were from well-described hospital-acquired lineages (*n*=23/86; 26.7%), including the globally distributed CC5 lineage (*n*=12) (Fig. 1a, b). Infections from this CC were often unresolved (*n*=6/12; 50%) and posed the highest risk of a ‘not cured’ patient outcome. CC22 is the most common ST identified from clinical infections, particularly in the UK [95,96], but was the third most sampled CC in our collection (*n*=11/86; 12.8%). This CC was implicated in a ‘not cured’ patient outcome on only one occasion. All other CCs were represented by fewer than five isolates. One isolate was isolated from the livestock-associated lineage, CC398 (*n*=1).

### Accessory genome differences in ODRI isolates

ODRI that leads to a ‘not cured’ patient outcome is a complex process, which is affected by genetic and environmental differences in the host (Table 2) as well as variation in the infecting bacterial population. To investigate differences in gene presence between isolates showing phenotypic variation, we further investigated genes in the accessory genome. We characterized the pangenome using PIRATE [97] with 86 isolates plus eight reference strains (to help preserve gene nomenclature). In total, PIRATE identified 4,142 gene clusters, of which over half were characterized as core genes (present in 95% or more of the isolates; 2,150 genes; 52%). This was consistent with core genome estimates from other *S. aureus* collections [98,101]. The accessory genome consisted of 1,992 genes (present in fewer than 95% of isolates), representing ~48% of the pangenome. A large proportion of the accessory genome (78%) was present in fewer than 25% of isolates (157 out of 1,992 accessory genes; Table S3). Core and accessory genome differences were visualized in phandango [75] (Fig. S1A). Consistent with the clonal nature of *S. aureus*, isolates grouped by accessory genome content (using PopPunk) clustered similarly to the clonal frame (Fig. S1B, C) [76].

### Distribution of known virulence and AMR genes

**Fig. 2.**
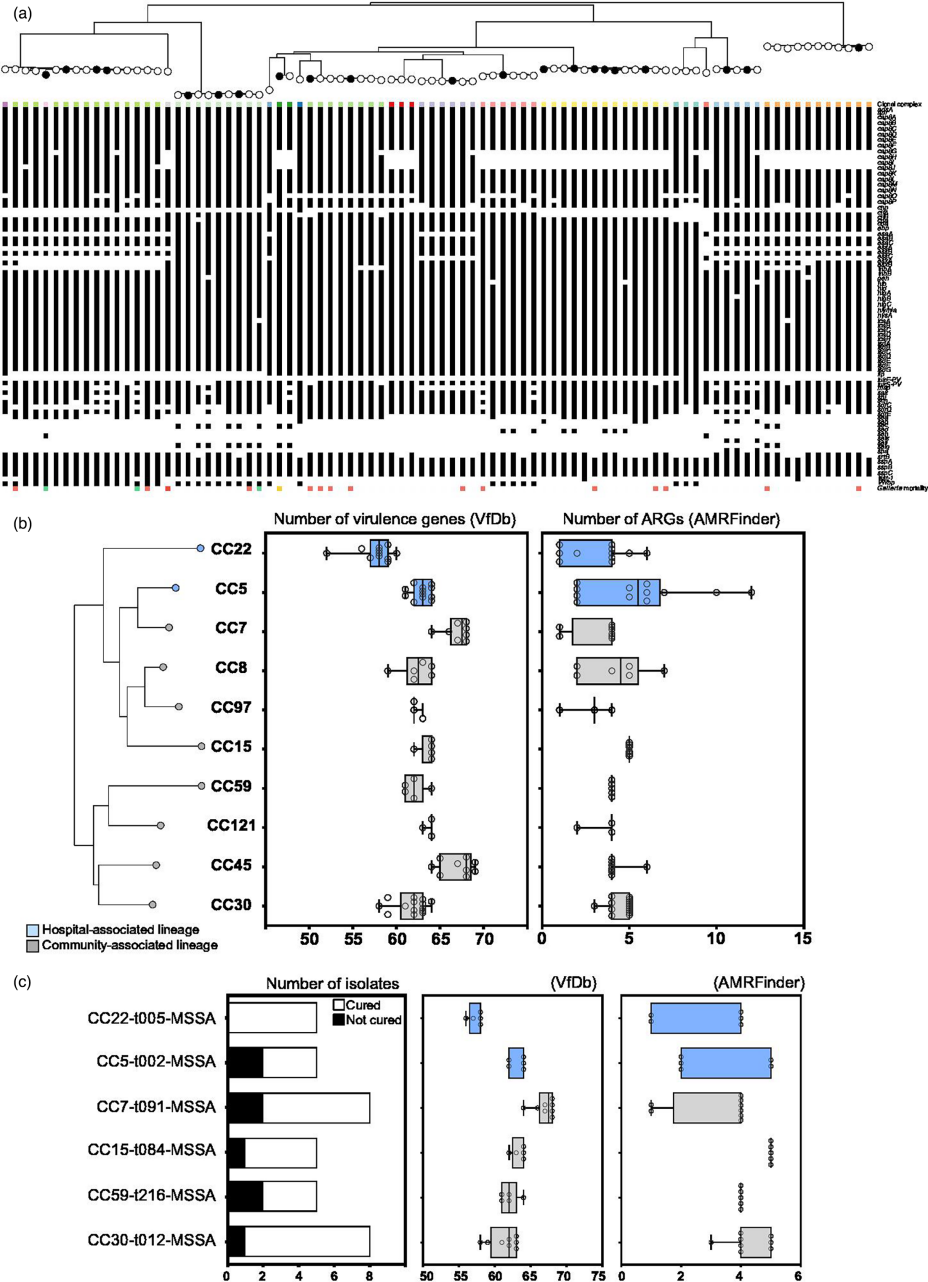
Presence of known virulence and antibiotic genes. (a) The same maximum-likelihood phylogeny of our 86 *S*. *aureus* isolates as Fig. 1 is visualized above the matrix of defined virulence factors identified using the VfDb [77]. Summary boxplots of the number of virulence and ARGs identified in each (b) CC and (c) MSSA clone (represented by three or more isolates). All data points (isolates) are shown, and bars show minimum and maximum values. Hospital-associated (HA) lineages and clones are highlighted in blue. The number of isolates from each MSSA clone is also shown, with the proportion of isolates from patients who did not achieve a ‘cured’ status coloured in black.

All *S. aureus* isolates were sampled from invasive disease cases and contained many known virulence genes, with between 50 and 69 genes identified in each isolate from the VfDB database (average: 63; update March 2021). Nearly half of these putative virulence genes were present in all isolates (35 of 79; 44%), including the genes: *adsA* (phagocytosis escape), *aur* (metalloproteinase), *geh* (lipase), *hla (α*-haemolysin), *hlgAB (γ-*haemolysin), *hysA* (hyaluronate lysate), *icaABDR* (biofilm formation), *isdAB* (surface proteins), *lip* (lipase), *srtB* (surface protein anchor), *sspABC* (adhesion) and much of the *cap8* capsule operon (Fig. 2a). Distribution of virulence genes between the two clinical outcome groups (‘cured’ and ‘not cured’) was also considered. Although generally rare in our collection, the methicillin resistance gene (*mecA*) was more prevalent in the ‘not cured’ outcome group (*n*=2/21; 9.5%) compared to the ‘cured’ group (*n*=3/65; 4.6%). Also, the presence of the *bbp* gene (bone sialoprotein binding) (*n*=20/21; 95.2% versus *n*=58/65; 89.2%) and *ebpS* gene (elastin binding) (*n*=21/21; 100.0% versus *n*=60/65; 92.3%) was found to be more prevalent in the ‘not cured’ outcome group than in the ‘cured’ outcome group, but this was not statistically significant (Table S4). The *scn* gene coding for the staphylococcal complement inhibitory protein (SCIN) was present in nearly all isolates (*n*=78/86; 90.7%) (Table S5). The distribution between ‘cured’ and ‘not cured’ outcomes was equal (*n*=59/65; 90.8% versus *n*=19/21; 90.5%). Based on the presence or absence of the five genes (*scn*, *chp*, *sak*, *sea* and *sep*), all isolates were typed into an IEC cluster type, except for eight isolates, which were non-typable due to the absence of the *scn* gene. IEC type B (*scn*, *chp* and *sak*) was the most common type (*n*=29/86; 33.7%), followed by type A (*scn*, *chp*, *sak* and *sea*) (*n*=18/86; 20.9%). IEC type E (*scn* and *sak*) was present in 12.8% (*n*=11/86) isolates, followed by the IEC types C (*scn* and *chp*) and D (*scn*, *sak* and *sea*) with *n*=10/86 (11.6%) isolates, respectively. Looking at the distribution within the outcome groups, the IEC types B and A (*n*=8/21; 38.1% and *n*=6/21; 28.6%, respectively) were more prevalent in the ‘not cured’ outcome group than in the ‘cured’ group, whilst IEC types C and D were more prevalent in the ‘cured’ outcome group (*n*=8/65; 12.3% versus *n*=2/21; 9.5%).

Few ARGs were identified through nt comparisons with the AMRFinderPlus database (Table S6). Differences were observed between CCs for the numbers of virulence and AMR determinants (Fig. 2b). In common with other studies, we identified slightly fewer virulence genes, but more ARGs in HA lineages. On average, there were 60.3 virulence genes and 4.4 ARGs in HA lineages, compared with 63.8 virulence genes and 4.1 ARGs in CA lineages. Six MSSA clones were represented by three or more isolates, of which five represented more than a third of our isolates (8 of 21; 38%) from patients who experienced a ‘not cured’ outcome (Fig. 2c). Two clones posed a particularly high risk to patients, with 40% (two of five) of CC5-t002-MSSA and CC59-t216-MSSA isolates developing a ‘not cured’ patient outcome. A quarter of patients (two of eight) infected by CC7-t091-MSSA also experienced a ‘not cured’ outcome, whilst those infected by the CC22-t005-MSSA clone all recovered.

### Both ‘cured’ and ‘not cured’ isolates induced high mortality in a *G. mellonella* model

**Fig. 3.**
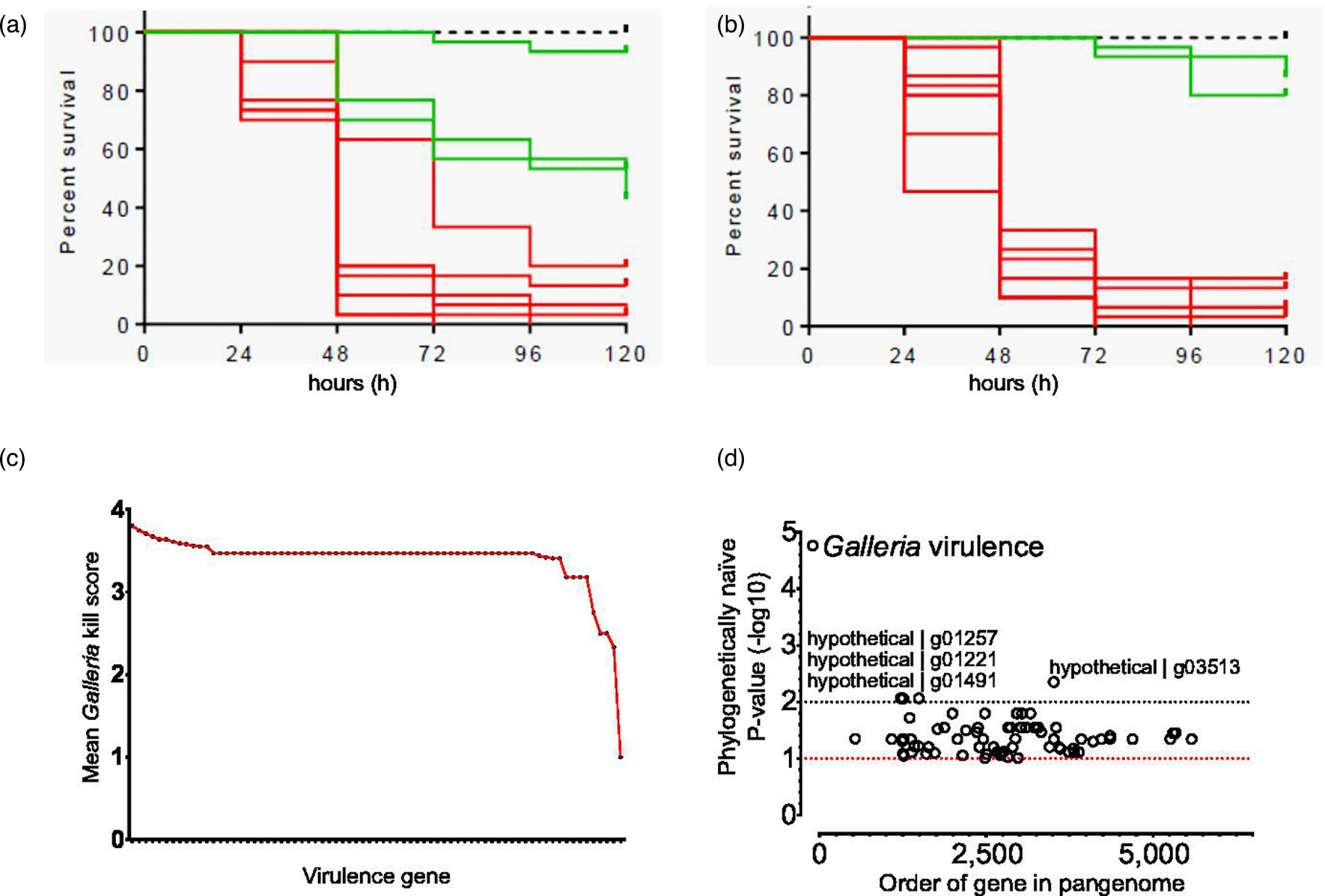
*G. mellonella* virulence model. The Kaplan–Meier survival curve of larvae infected with *S. aureus* isolates from (a) ‘not cured’ outcome patients and from (b) ‘cured’ outcome patients. Each line represents a different *S. aureus* isolate injected into ten larvae per isolate. The mean survival rate was calculated per isolate. The dotted line represents the survival of larvae injected with PBS, the green line represents a high survival rate and the red line indicates a high mortality rate. (c) Mean kill curve scores from isolates that contained each virulence factor identified by VfDb. (d) Pangenome-wide association study comparing isolates with high kill scores (above 50% at 120 h) vs those with low kill scores using Scoary [88]. No lineage correction was used, and the phylogenetically naïve minus log *P*-value was reported. Three genes from the previously identified pathogenicity island and the *msaC* gene from the *msaABCR* operon were associated with increased killing.

The *G. mellonella* model was used to provide a controlled model to assess virulence and to eliminate patient diversity and differences in infection treatment protocols. A random selection of isolates from a range of CCs (ten ‘not cured’ and ten ‘cured’) was used to challenge *G. mellonella* larvae. Bacterial suspensions were injected into larvae (average inoculum of 2.75×10^6^ cfu/larvae; range: 1.87×10^6^ to 4.29×10^6^) and incubated up to 120 h. However, high mortality was observed in isolates from both outcome groups (Fig. 3a, b). This may not be surprising as all isolates were from infection cases, and all were found to possess at least 12 toxin genes. Further analysis of the *Galleria* virulence results identified differences between the average mortality scores when specific putative virulence genes were present (Fig. 3c). Differences in gene content between isolates were quantified and scored using Scoary [88]. We identified four gene clusters associated with increased killing during the *Galleria* infection model (Fig. 3d). Three of which (g01257, g01221 and g01491) demonstrated more than 90% sequence similarity with an SOS response-activated pathogenicity island shared between *S. aureus* and *Staphylococcus epidermidis* isolates (SACOL0900-0904) [98,102]. The fourth gene cluster (g03516) identified was identical (100% nt similarity over 100% of the gene) to the *msaC* gene (SACOL1438/SAUSA300_1296). As an unencoded member of the *msaABCR* operon, this locus has an indirect role in the expression of virulence factors *aur*, *scp*, *ssp* and *spl*, which contribute to increased virulence and biofilm formation and trigger the onset of bacterial persistence [103,105].

### Isolated phenotypic variation may contribute to the onset of infection but does little to influence patient outcome

We tested laboratory phenotypes associated with increased virulence and compared these with patient outcomes. Individually, none of the phenotypes contributed significantly to a ‘not cured’ patient outcome (Table 3). Isolates were subject to antimicrobial susceptibility profiling (29 antibiotics), biofilm formation (Table S7), haemolytic activity and staphyloxanthin production (Table S8). Four isolates were considered MDR (resistant to three or more different classes of antibiotic). More than half (*n*=51/86; 59.3%) of all collected isolates were unable to form a thick biofilm under laboratory conditions. Furthermore, 61.6% (*n*=53/86) of all isolates produced staphyloxanthin, and 39.5% (*n*=34/86) showed haemolytic activity (Table 3), which is not further differentiated between potential toxin classes. Isolates that demonstrated haemolytic activity contribute to a ‘cured’ patient outcome (≥90% confidence; *P*=0.090, Table 3). Isolates that were able to produce a thick biofilm were often also able to produce staphyloxanthin (*n*=27/35; 77.1%; Table 4). However, poor biofilm-forming isolates that produced staphyloxanthin (*n*=26/51; 51.0%) were detrimentally associated with a ‘not cured’ patient outcome (*n*=15/26; 57.7%).

**Table 3.** Phenotypic risk factors

		Cured outcome				
*S. aureus* phenotype		No		Yes		*P*-value*
		*n*	(%)	*n*	(%)	
**Total**		21	24.4%	65	75.6%	
***Biofilm formation***						0.429
	No	14	27.5%	37	72.5%	
	Yes	7	20.0%	28	80.0%	
***Staphyloxanthin production***						0.976
	No	8	24.2%	25	75.8%	
	Yes	13	24.5%	40	75.5%	
***Haemolysis activity***						0.090
	No	16	30.8%	36	69.2%	
	Yes	5	14.7%	29	85.3%	
***Methicillin resistance***						0.598
	No	19	23.8%	61	76.3%	
	Yes	2	33.3%	4	66.7%	
***Aminoglycoside resistance***						0.077
	No	20	23.5%	65	76.5%	
	Yes	1	100.0%	0	0.0%	
***Multi-drug resistance***						0.139
	No	17	22.1%	60	77.9%	
	Yes	4	44.4%	5	55.6%	

*Chi-square test; *P*-value <0.05 was considered significant.

**Table 4.** Phenotype associations in combination with enhanced biofilm formation

		Cured outcome	
		No	Yes
		*n* (%)	*n* (%)
**Total**		21 (24.4)	65 (75.6)
**Biofilm formation**			
**Positive (*n*=35; 40.7%**)		7 (20.0)	28 (80.0)
	**Multidrug resistance**		
	Yes (*n*=5; 14.3%)	2 (40.0)	3 (60.0)
	No (*n*=30; 85.7%)	5 (16.7)	25 (83.3)
	**Methicillin resistance**		
	Yes (*n*=1; 2.9%)	0 (0)	1 (100)
	No (*n*=34; 97.1%)	7 (20.6)	27 (79.4)
	**Haemolytic activity**		
	Yes (*n*=10; 28.6%)	0 (0)	10 (100)
	No (*n*=25; 71.4%)	7 (28.0)	18 (72.0)
	**Staphyloxanthin production**		
	Yes (*n*=27; 77.1%)	4 (22.2)	14 (77.8)
	No (*n*=8; 22.9%)	2 (25.0)	6 (75.0)
**Negative (*n*=51; 59.3%)**		14 (27.5)	37 (72.5)
	**Multidrug resistance**		
	Yes (*n*=4; 7.8%)	2 (50.0)	2 (50.0)
	No (*n*=47; 92.2%)	12 (25.5)	35 (74.5)
	**Methicillin resistance**		
	Yes (*n*=5; 9.8%)	2 (40.0)	3 (60.0)
	No (*n*=46; 90.2%)	12 (26.1)	34 (73.9)
	**Haemolytic activity**		
	Yes (*n*=24; 47.1%)	21 (87.5)	3 (12.5)
	No (*n*=27; 52.9%)	25 (92.6)	2 (7.4)
	**Staphyloxanthin production**		
	Yes (*n*=26; 51.0%)	15 (57.7)	11 (42.3)
	No (*n*=25; 49.0%)	12 (48.0)	13 (52.0)

Note: Statistical analysis was not done due to a low sample size per group.

### Genes associated with different virulence phenotypes

**Fig. 4.**
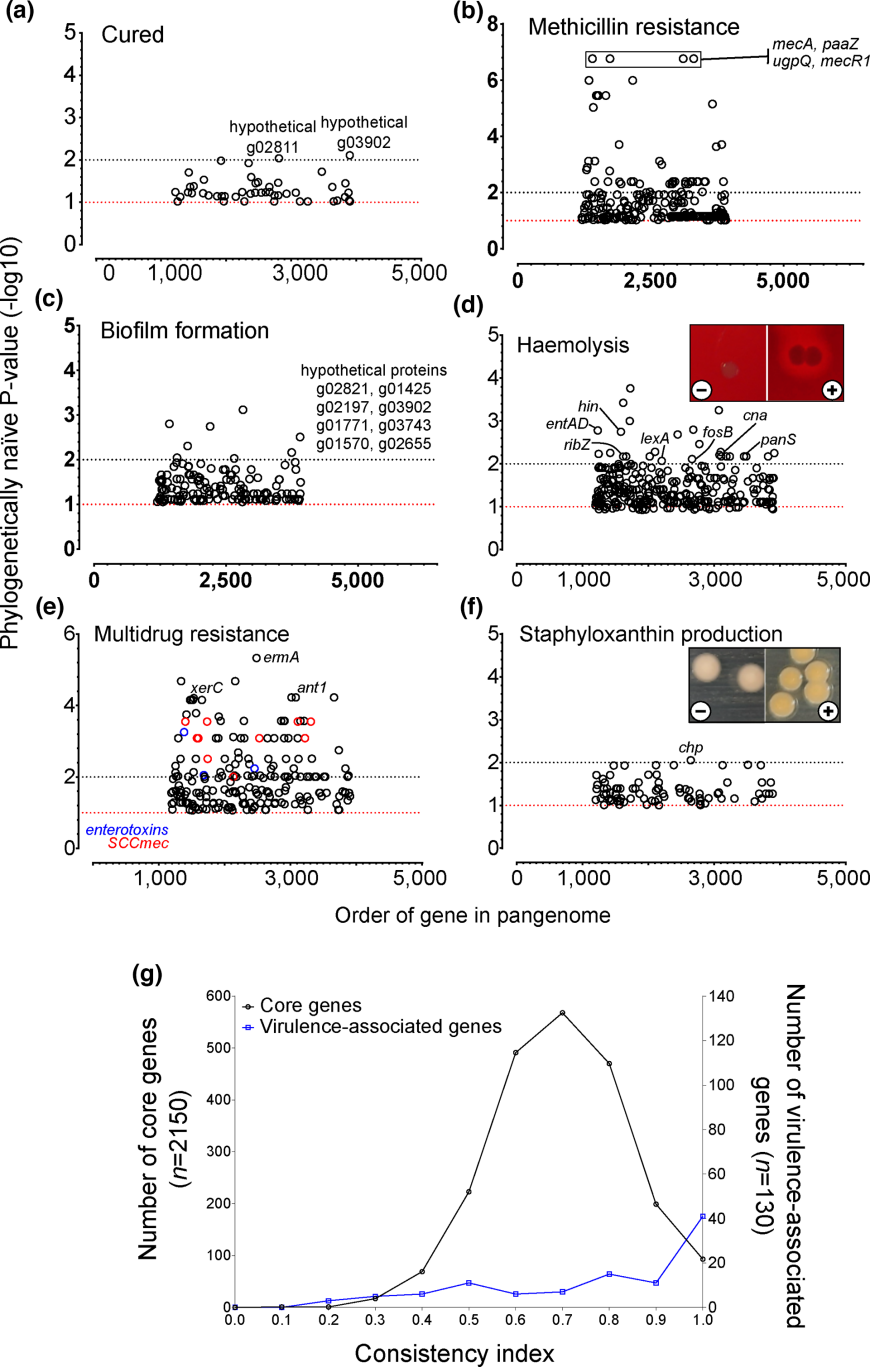
Pangenome-wide association studies. Scoary was used to quantify gene presence between isolates tested for six targeted virulence phenotypes [88]. No lineage correction was used, and the phylogenetically naïve minus log *P*-value was reported. Isolates were scored for phenotypic differences in (a) patient outcome, (b) methicillin resistance, (c) biofilm formation, (d) haemolysis, (e) multidrug resistance and (f) staphyloxanthin production. Genes that were highly associated with a given phenotype are labelled. Multiple toxin (blue circles) and SCC*mec* cassette (red circles) genes were associated with more than one phenotype. (g) Individual gene phylogenies were compared to a core phylogeny for each virulence phenotype-associated gene, and consistency indices were calculated. The two distributions were significantly different (Mann–Whitney test; U=78998, *P*<0.0001) between virulence phenotype-associated genes (0.7766±0.2418) compared with the average for all core genes (0.6943±0.1408).

Differences in gene content between phenotypes were quantified and scored using Scoary (Table S9). We identified genes associated with six different phenotypes, including patient outcome, biofilm formation, *β*-haemolysis, multidrug resistance, methicillin resistance and staphyloxanthin production (Fig. 4). An uncharacterized membrane protein (g02811) and a gene from the SA97 virulent bacteriophage (g03902) were associated with a ‘not cured’ patient outcome (-log_10_>2; naïve *P*-value <0.01; Fig. 4a). Amongst the small number of isolates in our collection that were MRSA (*n*=5), genes commonly found as part of the SCC*mec* cassette (*mecA*, *paaZ*, *upgQ* and *mecRI*) [106] were present in isolates that demonstrated methicillin resistance and were also amongst the gene clusters that demonstrated the strongest association with any phenotype (-log_10_>6; naïve *P*-value <2*10^−7^; Fig. 4b). Several hypothetical gene clusters were associated with biofilm formation (eight genes with naïve *P*-value <0.01; -log_10_>2; Fig. 4c).

The presence of several genes (27 genes with -log_10_>2, lowest *P*-value: 0.00017), including enterotoxins A and D and the collagen adhesin *cna*, was associated with haemolysis production (Fig. 4d). Despite only four isolates that were classed as MDR, the presence of 93 genes (lowest *P*-value: 4.68×10^−6^) was associated with phenotypically measured MDR, which included several members of the SCC*mec* cassette, the erythromycin resistance-associated *ermA* and the aminoglycoside resistance determinant, *ant1* (Fig. 4e). The MDR isolates also harboured several enterotoxin genes, along with the biofilm-associated gene *xerC*. The chemotaxis-inhibiting gene, *chp*, was the only gene associated with staphyloxanthin production in our dataset (Fig. 4f).

### Genes linked to phenotypes that influence patient outcomes are more recombinogenic

Finally, we investigated the role of recombination in isolates that led to a poorer patient outcome. On average, isolates that led to a ‘not cured’ patient outcome contained a higher number of single nucleotide variants (SNVs) imported by recombination than mutation (Fig. S2). However, genomes of isolates from patients who were successfully ‘cured’ were predicted to have included more recombination events. This is likely influenced by a small number of highly recombinogenic genomes that were predicted to have undergone extensive recombination (Table S10). Genes that were associated with any of our tested phenotypes demonstrated greater clonal inheritance than genes in the core genome, meaning that phylogenies constructed from sequences of the individual genes were consistent with the phylogeny constructed from the core genome (Fig. 4g). The mean consistency index was significantly higher (Mann–Whitney test; U=78998, *P*<0.0001) amongst virulence phenotype-associated genes (0.7766±0.2418) compared with the average for all core genes (0.6943±0.1408). Taken together, the highly clonal population structure of *S. aureus* (long branches) – particularly those genes involved in virulence – and higher rates of recombination between lineages permit parallel evolution of several successful lineages that can contribute to ‘not cured’ patient outcomes in ODRI.

## Discussion

Despite advances in many aspects of emergency and orthopaedic trauma care, ODRI persists as a challenge for the treating physicians and a significant burden for the patient. The interaction between the host immune system and invading isolates is complex and is affected not only by variation in host and bacterial genetics, but also by factors such as host well-being and general health (e.g. obesity). In a similar way to how the host deploys several different types of host defence mechanisms, bacteria are armed with a toolbox of different virulence factors. We used a combination of phenotype testing, an *in vivo* infection model and *in silico* genome characterization to identify lineages and virulence factors that may contribute to the risk of poor patient outcomes following ODRI.

Isolates from patients who experienced a ‘not cured’ outcome did not cluster by core or accessory genomes (Fig. 1a). The most common CC associated with a poor patient outcome in our collection was CC5, and 4 of 21 isolates from ‘not cured’ cases were CC5 (19%; Fig. 1b). CC5 is highly recombinogenic and able to infect multiple host species [107,108] and is often isolated from human infections, particularly skin and bone infections [99,109]. Whilst infections with CC5 isolates are often caused by MRSA, there is growing concern for the spread of virulent MSSA clones [109,110]. Nearly all the isolates that we collected were sensitive to methicillin and lacked a functional SCC*mec* cassette (9 of 12 CC5 isolates). The gain and loss of SCC*mec* cassettes by multiple *S. aureus* clones continue to blur the differentiation of community- (CA) and healthcare- (HA) associated *S. aureus* lineages [111,112].

Since the emergence and epidemic spread of the USA300 (CC8) clone in the early 2000s [37,94, 113], CA lineages have dominated infection surveillance studies [24,94, 113]. CA lineages are typically thought to carry larger – more cumbersome and less resistant – SCC*mec* types I, II and III and often carry toxin genes such as the PVL genes [114,115], whilst traditional HA *S. aureus* lineages carry smaller SCC*mec* cassettes that are more difficult to disrupt, and more often maintained by successive generations [114,115]. Several HA lineages rose to prominence around the world, only to have been replaced as the most common lineages in human infections in specific regions. This includes the rise of CC22 in the UK and Europe [23,116,118] and the replacement of HA-MRSA-ST239 by CA-MRSA-ST59 in China [24]. Increased virulence, coupled with fewer putative AMR genes, may have contributed to the success of these CA clones/lineages [38,119], and we observed a similar scenario in our collection – with more virulence genes and fewer ARGs found in CA lineages compared to HA lineages (Fig. 2b, Table S5).

Isolates from the most common European lineage (CC22) were common amongst our collection of invasive isolates and were the most likely to lead to a ‘cured’ patient outcome (Fig. 1b). Further characterization of the specific clones in our collection identified six common clones, including a CC22-t005-MSSA clone that did not lead to a ‘not cured’ patient outcome in any of the five patients it infected (Fig. 2c). The five remaining clones that were identified in three or more patients were responsible for more than a third of the ‘not cured’ patient outcomes. The CC59-t216-MSSA and CC5-t002-MSSA clones posed the highest risk, with 40% of patients infected by these clones developing a poor outcome. The CC5-t002-MRSA clone has been frequently observed, including studies from China and Iran where it exhibited extensive MDR, including mupirocin resistance, and contained several virulence toxins (PVL and TSST-1) [120,121].

All isolates that we collected were from invasive ODRI and contained at least 50 known virulence genes (Fig. 2a, b). Infection is complex, the function of any virulence gene is context-dependent and a simple sum of the number of putative virulence elements is unlikely to be a good proxy for the infective potential of an isolate. All isolates in our collection were able to form biofilms during colonization and cause an infection, but variation in additional virulence factors will influence disease progression and host evasion. Our understanding of the virulence potential of any given isolate may also be incomplete if genes involved in virulence or AMR are not included in reference databases. Genes can also be present and not expressed (and *in vitro* expression may not be representative of *in vivo* infection); or the full complement of genes required for a specific phenotype may not all be present.

Similar assays in *S. epidermidis* identified biofilm formation as key to establishing an ODRI through adhesion to implant surfaces [15,53] and contributed towards a poorer clinical outcome, persistence and recurrence [11]. However, this was not the case in the current study. Although 40% of the *S. aureus* isolates were able to form a strong biofilm, no clear influence on patient outcome was observed. Armed with a suite of extracellular toxins, *S. aureus* can often cause more severe acute infections [122], whilst *S. epidermidis* is often associated with less severe, chronic infection – where strong biofilm formation on implants and dead tissue may be more relevant [63,123, 124].

Grouping isolates by virulence-associated phenotypes yielded limited success, with an apparent mismatch between 40% (*n*=34 of 86) of isolates producing a clear ring of erythrocyte lysis around the colony, but nearly all isolates (99%; 85 of 86) containing a full complement of gamma-haemolysin genes (*hlgA-*C) (Table S4). To some extent, this can be explained by our assay, which detects haemolysis activity, but more specific assays would be required to determine the toxin types involved. *In vitro* staphyloxanthin production leads to increased pathogenicity of *S. aureus* [125,126], and 61% of our isolates were able to produce staphyloxanthin (Table S8). This level of prevalence is consistent with the observation by Post *et al.* whereby 56% of 305 isolates from implant and non-implant infections were staphyloxanthin producers [84]. The chemotaxis inhibitor protein (CHIPS), encoded by *chp*, was the only gene that we found associated with staphyloxanthin production (Fig. 4g). CHIPS’ ability to help evade host immune responses poses the potential for more severe and chronic infections [127,128], although it was not independently associated with ‘not cured’ in our study population. However, as CHIPS belongs to the immune evasion modulators, we observed that CHIPS together with the SCIN, staphylokinase and SE type A falling into IEC types B and A was more prevalent in the ‘not cured’ outcome group. This observation is in line with other studies reporting a high prevalence of IEC genes in clinical isolates, with type B being the predominant type [46,129].

As previously described, the dissemination of virulent MSSA lineages and clones was balanced with limited carriage of AMR genes, with relatively few isolates resistant to multiple antibiotics (Fig. 2b, Table S6), and lower rates of MRSA isolates compared to other studies (7% MRSA and 10% MDR). Overall, only one MDR isolate that resulted in a ‘not cured’ outcome was also methicillin-resistant. Rates of MRSA (27%–24%) were much higher in other studies of ODRI [13,14]. Both these previous studies involved patients with a median age above 60, and patients infected with MRSA experienced lower cure rates (57%) than for MSSA-infected patients (72%) or coagulase-negative staphylococci (82%) [13]. Very high rates of methicillin resistance have been observed in *S. epidermidis* ODRI isolates (73% MRSE and 76% MDR) [11]. Elderly patients are more likely to suffer from infection with resistant bacteria due to repeated hospital stays and multi-morbidity [130,131] and may partially explain the reduced number of MRSA isolates in our collection. Whilst we do not have specific data on patient age, due to the specialism of our study hospital (BMG Murnau), many of our patients were trauma patients receiving treatment for complex bone fractures. These patients are typically younger than most other patients who receive ODRIs [132]. Although only present in a small number of our isolates (*n*=6; 7%), methicillin resistance was associated with a ‘not cured’ outcome (two of six; 33.3%; Table 3). This is consistent with many other studies that have highlighted the detrimental effect of methicillin resistance on treatment success [53,99, 133].

Deep branching clades and highly structured clustering of isolates are evidence of the strong selection pressures that have driven the evolution of *S. aureus*. Distinct *S. aureus* lineages are found at markedly different prevalence globally, including virulent sub-lineages [117,134, 135]. Waves of pandemic sub-lineages have been described within CC30 – initially MSSA, before being replaced by MRSA variants [23,24, 117]. Independent acquisition of virulence determinants in these lineages was associated with the rise and fall of these lineages, and horizontal gene transfer likely has an important role in the accumulation of virulence factors 136. This lineage-specific accumulation of virulence genes makes traditional identification of virulence factors difficult. GWAS that can account for these lineage effects [53,54, 133, 137], or can differentiate between the types of association, are potentially useful [138]. Furthermore, the inclusion of the pangenome analyses (including mobile elements and intergenic regions) and covarying SNVs and genes (epistasis) [139,140], combined with appropriate phenotypic validation and/or transcriptome profiling, will greatly enhance the understanding of the contribution of pathogen genetic variation on disease progression [59].

This study provides valuable insights into the virulence-associated characteristics of ODRI isolates, although there are some important limitations to consider. Whilst the sample size was relatively small compared to larger epidemiological studies, it was sufficient to identify key bacterial traits linked to virulence. A major strength of this study is the inclusion of linked patient follow-up data, which distinguishes it from other studies that often lack outcome information. However, clinical factors such as surgical management, antibiotic duration and the need for additional procedures, which are known to significantly impact treatment outcomes, were not fully controlled for. To manage sample size, we grouped isolates and patients into broader clinical categories, but future studies with larger cohorts and more detailed stratification could provide deeper insights into how these clinical variables influence infection outcomes. The associations between bacterial traits and clinical outcomes identified in this study are promising, but larger, more diverse cohorts are needed to better account for these factors and strengthen our findings.

In addition, incorporating colonizing isolates or those from non-bone/joint infections could provide a broader context for understanding strain-specific adaptations. The 2-year follow-up period offers a solid foundation for tracking infection recurrences, and most *S. aureus* infections typically manifest within this timeframe. However, longer follow-up could help capture late recurrences. Whilst the *G. mellonella* model has limitations in replicating human infection dynamics, it remains a valuable tool for early-stage virulence testing. Future studies using more complex mammalian models will help validate these findings. Overall, this study lays the groundwork for future research, offering key insights that can be expanded upon with larger, more detailed datasets that integrate both bacterial and clinical factors.

Infection is clearly a complex process influenced by the host immune system and genotypic variation within the invading pathogen. In addition, patients receive both antibiotic therapy and surgical debridement, which can also influence outcome and at least partially overcome bacterial factors and their influence on outcome. Here, we only tested a single *S. aureus* colony from each patient. However, there can be considerable diversity amongst the commensal and infective isolates within a single person [141,144]. Small colony variants [145,146], persistence [147,148] and dormancy [149,150] also help protect invading cells from the host immune system and evade antimicrobial treatment. Whilst the isolates collected here have already colonized host subcutaneous tissue and established an infection, our aim was to delineate factors that contribute to poorer patient outcomes. Our results are consistent with a complex balance between virulence and colonization [39], involving core and accessory genome elements, and provide some evidence that characterization of common emerging clones and lineages may help disease outcome prediction.

## Supplementary material

10.1099/mgen.0.001390 Uncited Supplementary Material 1.

10.1099/mgen.0.001390 Uncited Supplementary Material 2.

## Abbreviations

AMR: antimicrobial resistance
ARG: antibiotic resistance gene
CA: community-associated
CA-MRSA: community-associated MRSA
CARD: comprehensive antibiotic resistance database
CCs: clonal complexes
cfu: colony forming unit
CHIPS: chemotaxis inhibitor protein
FRI: fracture-related infection
GTR: general time reversible
GWAS: genome-wide association study
HA: hospital-associated
IEC: immune evasion cluster
MDR: multidrug-resistant
MIC: minimum inhibitory concentration
MLST: mutilocus sequence typing
MRSA: methicillin-resistant *Staphylococcus aureus*
MSSA: methicillin-susceptible *Staphylococcus aureus*
OD: optical density
ODc: optical density cut off
ODRIs: orthopaedic device-related infections
PBS: phosphate-buffered saline
PJIs: peri-prosthetic joint infections
PVL: Panton–Valentine leukocidin
SCC*mec*: staphylococcal cassette chromosome *mec*
SCIN: staphylococcal complement inhibitory protein
SEs: staphylococcal enterotoxins
SNV: single nucleotide variant
STs: sequence types
TSA: tryptone soy agar
TSB: tryptone soy broth
TSST: toxic shock syndrome toxin
VfDb: virulence finder database
