# Supplementary material for: Methicillin-sensitive Staphylococcus aureus lineages contribute towards poor patient outcomes in orthopaedic device-related infections
Source: Microb Genom. 2025 Apr 16;11(4):001390. doi: 10.1099/mgen.0.001390 (PMC12068410; doi:10.1099/mgen.0.001390)
Supplement: Uncited Supplementary Material 1. [file mgen-11-01390-s001.pdf]

Supplementary material for the manuscript:

Methicillin-sensitive Staphylococcus aureus lineages contribute towards poor patient outcomes in orthopaedic device-related infections

Virginia Post[1], Ben Pascoe[2,3]*, Matthew D. Hitchings[4], Christoph Erichsen[5], Julian Fischer[6], Mario Morgenstern[7], R. Geoff Richards[1], Samuel K. Sheppard[2#] & T. Fintan Moriarty[1,7#]*

[1]AO Research Institute Davos, Davos, Switzerland
[2]Ineos Oxford Institute of Antimicrobial Research, Department of Biology, University of Oxford, Oxford, United Kingdom
[3]Faculty of Veterinary Medicine, Chiang Mai University, Chiang Mai, Thailand
[4]Swansea University Medical School, Swansea University, Swansea, United Kingdom
[5]Department of Trauma Surgery, Trauma Centre Murnau, Murnau, Germany
[6]Centrum of Orthopedic Isartal, Pullach im Isartal, Germany
[7]Department of Orthopedic and Trauma Surgery, University Hospital Basel, Switzerland

Running title: Orthopaedic device related S. aureus infections

#**Address correspondence to**: T. Fintan Moriarty, fintan.moriarty@aofoundation.org or Samuel K. Sheppard, samuel.sheppard@biology.ox.ac.uk

**VP and BP contributed equally to this work. SKS and TFM contributed equally to this work.*

Table of contents

3 **Figure S1:** Accessory genome characterisation.
4 **Figure S2:** Recombination analysis.

[separate .xlsx file]

Table S1: Demographic data collected for each isolate.
Table S2: Genome quality control and extended typing results.
Table S3: Summary of core and accessory genome characterization with PIRATE.
Table S4: Summary of genes identified by screening known virulence genes with the VfDB.
Table S5: Summary of IEC genes and typing.
Table S6: Summary of genes identified by screening antibiotic resistance genes with the AMRfinder (NCBI) and CARD databases.
Table S7: Summary of OD values of biofilm assay.
Table S8: Summary of virulence phenotype data.
Table S9: Summary of genes associated with virulence phenotypes (SCOARY).
Table S10: Per isolate recombination statistics using Gubbins.

Additional materials and files

Figshare: All supplementary files can also be found on FigShare doi: 10.6084/m9.figshare.7926866

Microreact: Interactive phylogenies are hosted with [https://microreact.org/project/1KsjRuuiKzsuioCxQMc1HF-davos-odri-s-aureus-virulence]
and without AMR and virulence determinants [https://microreact.org/project/u7RjbMpReHLikTWN8Angxf-postpascoeetal-saureus-odri]

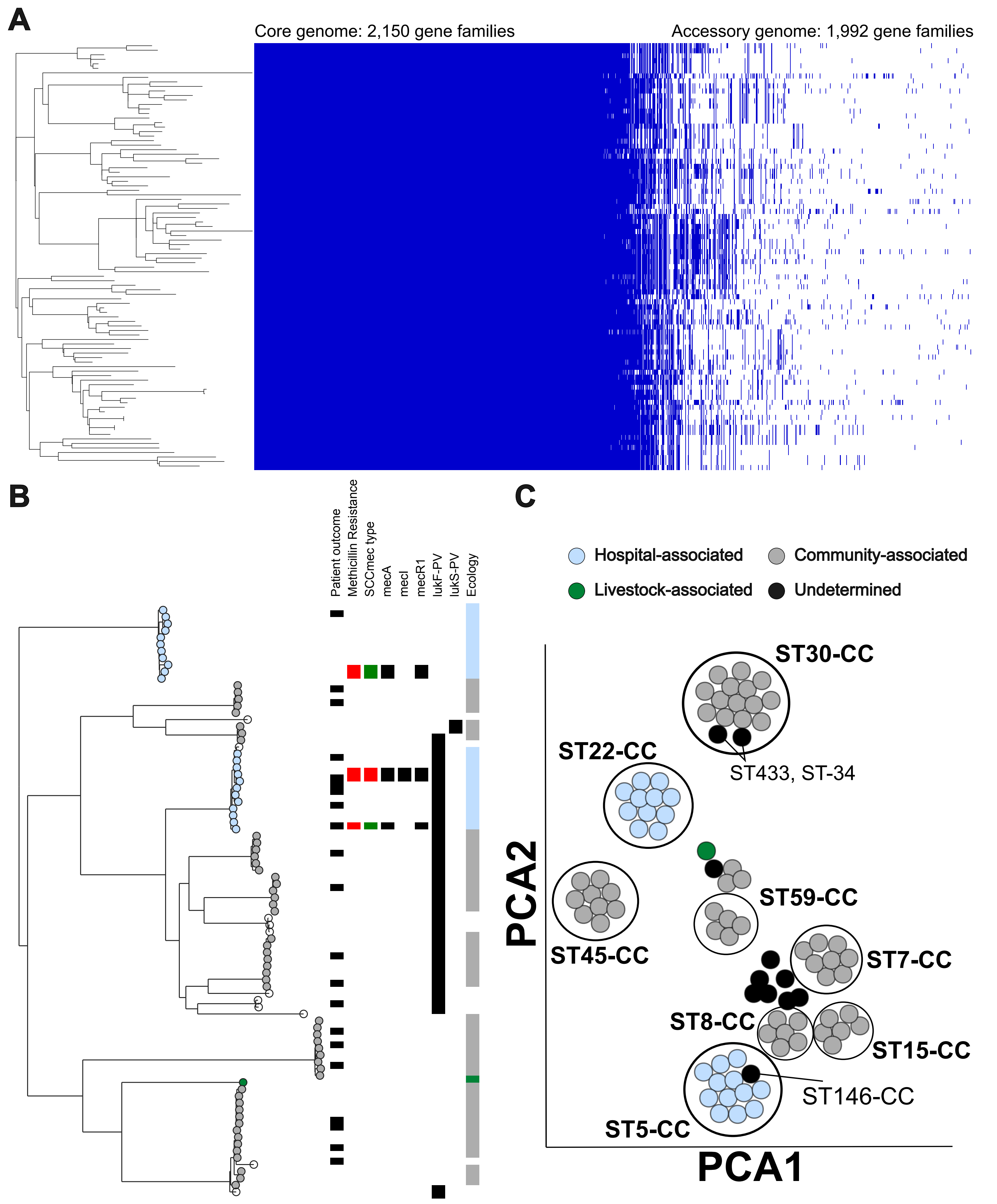

Supplementary figure S1: (A) Pangenome matrix from PIRATE visualised in phandango. Blue blocks represent shared gene families between isolates. (**B**) Alternative visualisation of the same maximum likelihood phylogeny of our 86 isolates. Isolates are coloured by their predicted clonal lineage origins; hospital-, community-, livestock-, and undeterimed lineages are coloured in blue, grey, green and black, respectively. (**C**) Accessory genome clustering of isolates with PopPunk groups isolates similarly to their position in the core genome phylogeny.

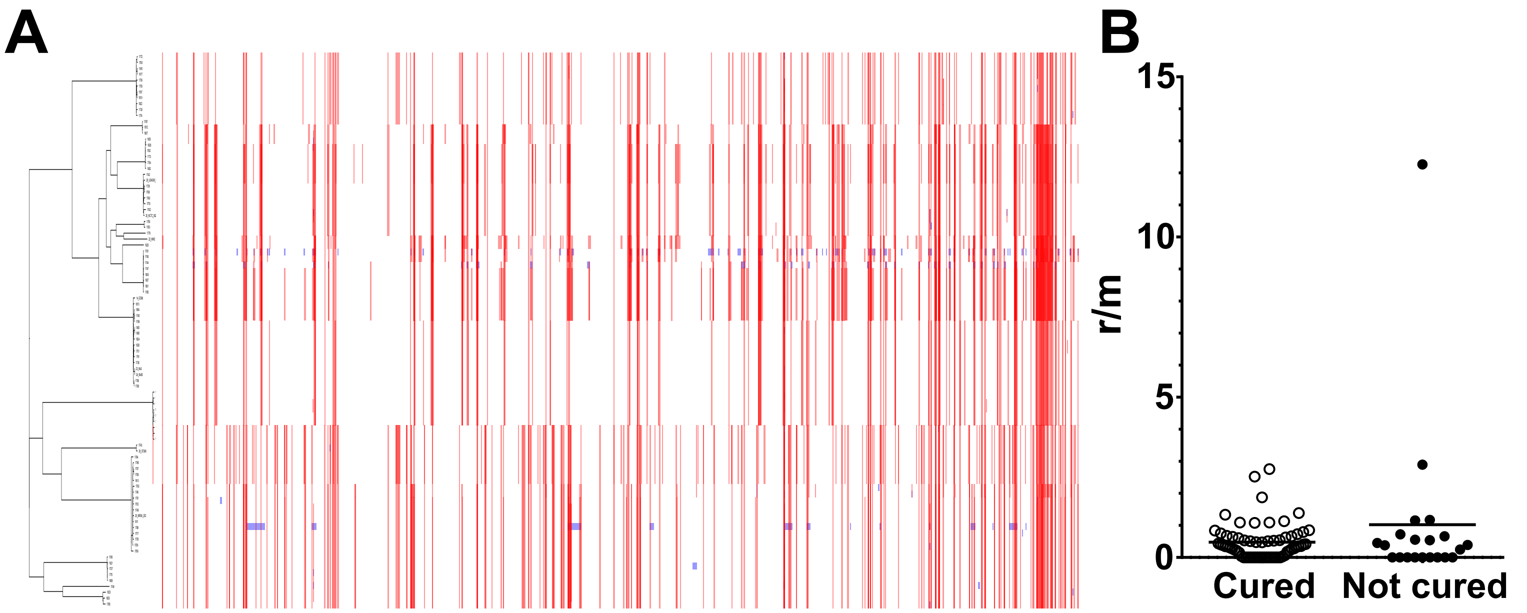

Supplementary figure S2: (A) The same maximum likelihood phylogeny of our 86 isolates as Figure 1 is visualised in phandango alongside output from Gubbins. Red lines represent areas of putative recombination events that are common in multiple isolates, and blue areas are those identified only in a single isolate. The effective rate of recombination (r/m) for each isolate, grouped by infection outcome ("cured" / "not cured") is summarised (**B**). Each circle represents an isolate and the average rate is designated by a black line. The effective rate of recombintaion (r/m) is measured by the frequency at which recombination (r) occurs relative to mutation (m).
